# Isoflavonoids – an overview of their biological activities and potential health benefits

**DOI:** 10.2478/v10102-009-0021-3

**Published:** 2009-12-28

**Authors:** Eva Miadoková

**Affiliations:** Department of Genetics, Faculty of Natural Sciences, Comenius University, Bratislava, Slovak Republic

**Keywords:** isoflavonoids, phytoestrogens, health benefits, mechanisms of action

## Abstract

There are many biological activities attributed to isoflavonoids. The majority of them could be beneficial and some of them may be detrimental, depending on specific circumstances. Isoflavonoids play an important role in human nutrition as health promoting natural chemicals. They belong to plant secondary metabolites that mediate diverse biological functions through numerous pathways. They are structurally similar to estrogens, exerting both estrogenic and antiestrogenic properties in various tissues. The results of epidemiologic studies exploring the role of isoflavonoids in human health have been inconclusive. Some studies support the notion of a protective effect of their consumption in immunomodulation, cognition, risk reduction of certain cancers, cardiovascular and skin diseases, osteoporosis and obesity, as well as relief of menopausal symptoms. Other studies failed to demonstrate any effects.

## Introduction

Interest in possible health benefits of isoflavonoids has increased due to a variety of bioprotective effects, including antioxidant, antimutagenic, anticarcinogenic, antiproliferative activities, mostly assessed *in vitro* (Birt *et al*., 2001; Miadokova *et al*., 2002; Ryan-Borchers *et al*., 2006; Iwasaki *et al*., 2008; Scarpato *et al*., 2008). Isoflavonoids have been classically defined as dietary antioxidants, i.e. compounds that may protect against oxidative stress linked to inflammation and the risk of macromolecule damage by free radicals and by related oxygen and nitrogen-based oxidizing agents (Reiter *et al*., 2008). They may protect the body from hormone-related cancers, like breast, endometrial (uterine) and prostatic. Despite the wide spectrum of health protective abilities that have been attributed to them, e.g. immunomodulation, risk reduction of chronic diseases including cardiovascular diseases, diabetes, cancer, osteoporosis and obesity (Bezek *et al*., 2008), as well as relief of menopausal symptoms (Birt *et al*., 2001; Ryan-Borchers *et al*., 2006; Ørgaard and Jensen, 2008; Sabudak and Guler, 2009), there is growing evidence from human dietary and epidemiologic studies that the role of isoflavonoids in human health is questionable. Some studies speak in favor of a protective effect of isoflavonoid consumption as dietary supplements and as a natural alternative to estrogen replacement therapy, other studies failed to demonstrate favorable effects. The aim of this article is not to present a comprehensive review of the literature but to provide a critical review of the arguments centered on a variety of effects and mechanisms of isoflavonoid actions.

## Natural sources, bioavailability and metabolism

Isoflavonoids, as plant secondary metabolites, possess a 3-phenylchroman skeleton that is biogenetically derived from 2-phenylchroman skeleton of flavonoids. Isoflavonoids are particularly prevalent in *Papilonoidae*, subfamily of *Leguminosae* (Dixon and Summer, 2003). Soy (*Glycine max*) principle isoflavones genistein, daidzein and glycitein are the aglycones with three possible glycoside forms: β-D-glycoside, 6″-O-malonyl-glycoside and a 6″-O-acetyl-glycoside. However, primarily they are naturally present in their β-D-glycoside form as genistin, daidzin, glycitin. The aglycone form of isoflavones is biologically active. In addition to soy, isoflavone daidzein and genistein, their precursors formononetin, biochanin A, their glycosides, glycosides malonates and acetyl glycosides were determined in red clover (*Trifolium pratense*) extracts using chromatographic and spectrometric methods (Sabudak and Guler, 2009). Murata *et al*. (2006) isolated from leaves of *Millettia taiwaniana* (*Leguminosae*) two new isoflavonoids, millewanin-F and furowanin-A, together with previously known five isoflavonoids. The bioavailability of isoflavones has been shown to be influenced by their chemical form in foods (generally glycoside conjugates), susceptibility to degradation, the microbial flora of the consumer, and by the food matrix. Bioavailability of isoflavonoids may depend to some extent upon interaction with other dietary components (Birt *et al*., 2001).

The metabolism of isoflavones in animals and humans is complex and is a combination of mammalian and gut microbial processes. Moreover, there is a great deal of individual variability in the metabolism of isoflavonoids. Individual differences in gut microflora, intestinal transit time, and genetic polymorphisms, all are likely to contribute to this great variability (Duffy *et al*., 2007). During digestive and absorptive processes, isoflavonoids often undergo metabolic transformations. In some individuals, daidzein can be converted by the intestinal microflora to the metabolite equol or to O-desmethylangolensin, and genistein to p-ethyl phenol.

The degree of absorption of bioflavonoids has been the subject of frequent debates among scientists. Absorption and bioavailability of isoflavonoids is much higher than originally believed. Cesarone *et al*. (2009) compared absorption of an isotonic isoflavonoid solution vs. tablet form with the equivalent amount of fluid and revealed a dramatically accelerated bioavailability of isoflavonoids delivered in an isotonic formulation.

Under certain nutritional conditions total isoflavone concentrations can be measured in urine, plasma and even in breast fluid. Assessment of isoflavonoids in nipple aspirate fluid enables to elucidate their effect directly on breast tissue (Maskarinec *et al*, 2008a).

The hypothesis that isoflavones are absorbed more efficiently from fermented than from non-fermented soy foods was re-examined and then rejected (Maskarinec *et al*., 2008b). Therefore recommendations favoring fermented soy foods are not justified when the intestinal microflora is capable of hydrolyzing the isoflavone glycosides from non-fermented soy foods.

## Estrogenic activity of isoflavonoids

Isoflavones are diphenolic compounds that are structurally or functionally similar to endogenous estrogens and display agonistic and antagonistic interactions with estrogen receptors (Wang *et al*., 2008). Their biological activity is partly ascribed to the structural similarities with the primary physiologically relevant estrogen – 17β-estradiol (E2). They bind to and activate intracellular estrogen receptors: ERα and ERβ and, mimicking the effects of estrogen, are commonly referred to as phytoestrogens. In most systems, the relative binding affinities of genistein and daidzein are greater for ERβ than for ERα, while E2 binds to both receptors with approximately equal affinities (Messina *et al*., 2006). The estrogenic activities of soy isoflavones are thought to play an important role by their health-enhancing properties in menopausal symptoms and in treating osteoporosis (Lockwood, 2008). Though isoflavonoids have exhibited estrogen-like properties they are bound more weakly to estrogen receptors than E2. Arguments have been presented for considering soy isoflavones as natural selective estrogen modulators.

## Controversal data on estrogen-like effects

Since overexposure to estrogen is a major contributing factor in the development of breast cancer, the relationship between soyfoods and breast cancer has become controversial. A concern has been raised that soy-derived isoflavones, which exhibit estrogen-like properties under certain experimental conditions, may stimulate the growth of existing estrogen-sensitive tumors. This concern exists because of evidence showing that isoflavones bind and transactivate estrogen receptors and elicit estrogenic effects in rodent reproductive tissues. Limited human data directly address the tumor-promoting effects of isoflavonoids (Messina *et al*., 2006)

The fact that genistein at low physiologically relevant levels may stimulate estrogen receptor positive tumors can be attributed to its estrogenic properties, while at higher levels, anticancer actions of isoflavonoids may predominate (Duffy *et al*., 2007).

Estrogen-like effects have raised concern regarding soy/isoflavone consumption particularly in the case of postmenopausal women at high risk of breast cancer. Currently there is little evidence to suggest that any potential weak estrogenic effects of dietary isoflavones have a clinically relevant impact on the breast tissue in healthy women or breast cancer survivors (Messina and Wood, 2008).

Epidemiologic evidence shows that higher soy intake in Asian women is associated with a nearly one-third reduction in breast cancer risk and that Japanese breast cancer patients, in comparison to Western women, exhibit better survival rates even after controlling stage of diagnosis. Evaluation of the short-term effects of high-dose soy isoflavonoid supplements on reproductive tissues in a postmenopausal primate model showed that high doses of dietary isoflavonoids had minimal uterotrophic or mammotrophic effects (Wood *et al*., 2006). These findings suggest that comparable high-dose isoflavonoid supplements, in particular those containing equol or taken by equol producers, would have minimal negative estrogenic effects on human reproductive tissues and are not likely to contribute to increased uterine or breast cancer risks.

Since endogenous estrogens are important determinants of breast cancer risk in postmenopausal women, Wood *et al*. (2007) evaluated the effects of dietary soy isoflavonoids on endogenous metabolism in a postmenopausal primate model. They revealed that isoflavonoid treatment did not significantly alter gene markers of estrogen metabolism or estrogen receptor agonist activity in breast tissue.

On the other hand, there are data supporting the idea that a high dietary isoflavone intake improves the prognosis of breast cancer patients. For example, among postmenopausal U.S. breast cancer patients, mortality was reduced due to isoflavonoid diet (Fink *et al*., 2007). Available data for estrogen therapy effects on breast cancer recurrence and mortality provide some assurance for breast cancer patients that isoflavone supplements, when taken at dietary levels, do not contribute to recurrence rates. Nevertheless more data are needed to better address this issue (Messina and Wood, 2008).

## Phytoestrogens and effects on gene expression

Isoflavones can exert hormonal and nonhormonal properties in many ways. Isoflavones have been shown to inhibit the activity of enzymes involved in estrogen metabolism. Estrogens have diverse effects throughout the body, attributable in part to their ability to modulate transcription of target genes in a variety of organs. Rice *et al*. (2006) found that phytoestrogens and their low dose combinations inhibited mRNA expression and activity of aromatase in human granulosa-luteal cells. Due to the established association between estrogen levels and breast cancer risk, the inhibitory effect of biochanin A on enzyme aromatase, the protein product of *CYP 19* gene, was studied. Investigation of the effect on gene regulation and enzyme activity of aromatase showed that this isoflavonoid inhibited *CYP19* expression and aromatase activity and hampered the growth of MCF-7 breast carcinoma cells attributed to the enzyme activity (Wang *et al*., 2008).

At nutritionally relevant doses, phytoestrogens may selectively interact with ERβ and thus affect only expression of a subset of estrogen-responsive genes. Different regulation of estrogen-responsive genes by isoflavonoids may also depend upon the relative ratio of ERα and ERβ present within tissue types. To test the hypothesis that soy isoflavones selectively trigger Erβ-dependent gene expression, which may be particular to the cell type and dependent on relative expression of ERα and Erβ, Chrzan and Bradford (2007) studied whether genistein and daidzein could affect reporter gene transcription via the estrogen receptors ERα and ERβ1. They documented that both phytoestrogens, genistein and daidzein, increased the expression of estrogen-responsive genes in human MCF-7 breast carcinoma cells.

Data from experiments using DNA microarray analysis for examining the effects of genistein in the developing rat uterus indicate that genistein alters the expression of 6–8 times as many genes as does physiological estrogen E2. Genistein affected 227 genes, the majority of which were down regulated (Barnes, 2004).

Emerging research using genomics and proteomics enabled to understand the participation of enzymes included in the isoflavonoid metabolic pathway regulation. Ralston *et al*., (2005) cloned and functionally characterized soybean chalcone isomerases (CHIs), key enzymes in the phenylpropanoid pathway that produces flavonoids and isoflavonoids. Gene expression and kinetics analyses revealed that the gene expression for soybean type I CHI, which uses naringenin chalcone as substrate, is coordinately regulated with other isoflavonoid-specific genes, while the legume specific type II CHIs, which uses a variety of chalcone substrates, are coordinately regulated with an isoflavonoid-specific gene and specifically activated by nodulation signals. Comparison of putative soybean CHI with CHI ortologs from other species showed that their amino acid sequences fell into four different subfamilies. CHIs in the first subfamily (CHI1) were found only in legumes and were similar at least in 70% to those in Plant Genome Database (Ralston *et al*., 2005).

## Inconsistency between phytoestrogen intake and risk for colorectal cancer

Some experimental studies reported that anticarcinogenic properties of dietary soy isoflavonoids play an important role in preventing colorectal cancer. However, few epidemiologic studies have examined this association in general populations and their findings have been inconsistent (Akhter *et al*., 2008).

Investigation of the association between dietary isoflavone intake and incidence of colorectal cancer in a prospective cohort study of 83,063 Japanese men and women documented that the intake of isoflavones was not associated with the risk of distal colorectal and rectal cancers in either men or women. Moreover, the risk of proximal colon cancer in men decreased with increasing consumption of isoflavones, miso soup and soy food (Akhter *et al*., 2008). These findings are in accordance with those aimed at soy isoflavones modulation of rat colon carcinogenesis because pre- and postnatal exposure to dietary soy isoflavones suppressed the growth of colon tumors in male rats (Raju *et al*., 2009).

## Inconsistency between phytoestrogen intake and risk for breast cancer

Data regarding the role of isoflavonoids in breast cancer prevention are conflicting. Nevertheless, they suggest that early exposure in childhood or early adolescence may be protective (Ju et al., 2006; Duffy *et al*., 2007). There has been some evidence suggesting that the menopausal status of women may modulate the effect of isoflavonoids. Case-control studies examining phytoestrogens and breast cancer incidence have generally found more evidence for a protective role in premenopausal women versus postmenopausal ones because isoflavonoid effects are dependent on the hormonal status of the woman, with stimulatory effects in low-estrogen environments, while in high-estrogen states they may block the effects of estrogen.

There are *in vivo* animal data suggesting that genistein may interfere with inhibitory effects of tamoxifen on breast cancer cell growth (Liu *et al*., 2005). Several studies using urine serum concentrations of phytoestrogens and their metabolites as biomarkers of their intake reported reduced risk for breast cancer among breast cancer survivors, yet other studies have failed to show an effect of phytoestrogens on breast cancer risk or on the menopausal syndrome (Duffy *et al*., 2007). Recently Goodman *et al*. (2009) examined the association of urinary phytoestrogens with the risk of postmenopausal breast cancer and results of their multiethnic cohort study revealed that a diet rich in isoflavones from soy products reduced the risk of postmenopausal breast cancer, particularly in populations with comparatively high excretion of phytoestrogens. Iwasaki *et al*., (2008) also found a statistically significant inverse association between plasma genistein and the risk of breast cancer.

Some differences in the results presented could be related to other abettors, such as genetic factors which may modulate the effect of phytoestrogens (Piller *et al*., 2006). Functional polymorphism in genes that encode for enzymes involved in the estrogen biosynthesis and metabolism and in genes that encode hormone receptors may have been associated with the risk for breast cancer (Atkinson *et al*., 2004). But no association between a polymorphism in genes *CYP17* (5′untranslated *Msp*A1 polymorphism), *CYP19* (generated by a G → T substitution in intron 6), involved in the sex hormone biosynthesis pathway, and *Pvull* (lipoprotein lipase) polymorphism (generated by a C → T substitution in intron 1) in the estrogen receptor *ESR1* gene and the risk of breast cancer was demonstrated (Chen *et al*., 2008). Piller *et al*. (2006) examined dietary genistein intake, *CYP17* 5′untranslated *MspA1* genetic polymorphism and breast cancer risk in premenopausal breast cancer patients. The risk-reducing effect of genistein consumption was not modified by *CYP 17* genotype. On the other hand, examination of two single nucleotide polymorphisms in *Cyp 19* gene (aromatase), *rs1008805* (A/G) and *rs730154* (C/T) revealed that premenopausal women carrying at least one *G* alelle at *Cyp 19* locus was associated with an increased breast cancer risk (Talbot *et al*., 2008). Furthermore, *Cyp17* variant *C* alelle may increase the breast cancer risk in conjugation with long-term hormone replacemet treatment (HRT) and a high body mass index (BMI) in postmenopausal women (Chen *et al*., 2008). These findings support the potential role of variation in estrogen biosynthesis genes in premenopausal and postmenopausal breast cancer risks.

Additionally, results obtained by Boccia *et al*. (2005), proved that *SULTA1Arg213His* gene (encoding sulfotransferase) polymorphism is a potential marker for identifying gastric cancer individuals at a high-risk, and useful in defining who may benefit from specific chemopreventive interventions.

## Proposed mechanisms for cancer prevention

Isoflavonoids have been shown to possess many biological properties that may account for cancer prevention. Isoflavones exert their effects through numerous pathways and with respect to cancer prevention, they use mechanisms of action which appear to be various, complementary and/or overlapping.

Some mechanisms of action have been identified for isoflavone/flavone prevention of cancer, including estrogenic/antiestrogenic activity, antiproliferation, induction of cell cycle arrest and apoptosis, prevention of oxidation, induction of detoxification enzymes, regulation of host immune system and changes in cellular signaling (Birt *at al*., 2001; Murata *et al*., 2006; Chrzan and Bradford, 2007). It is expected that also combinations of these mechanisms may contribute to cancer prevention.

Dysregulated proliferation appears to be a hallmark of an incerased susceptibility to neoplasia. Cancer prevention is generally associated with inhibition, reversion or retardation of cellular hyperproliferation. It is known that dietary isoflavonoids may behave as general cell inhibitors. Although most isoflavonoids appear to be non-toxic to humans and animals, they have been shown to inhibit proliferation, the cell cycle or induce apoptosis in many kinds of cultured cancer cell lines. It was thus found that check points at both G1/S and G2/M of the cell cycle were perturbed by isoflavonoids (Birt *at al.,* 2001).

Soy-protein-derived isoflavonoids have antiproliferative and proapoptic effects on prostatic epithelial cells *in vitro* and on the prostate gland of macaques, preventing prostate cancer development and/or progression in animal populations consuming soy protein (Hedlund *et al*., 2005; Perry *et al*., 2007). Activation of Erβ is supposed to result in decreased proliferation due to the receptor expression loss because Erβ probably functions as tumor suppressor within the prostate gland. The Erβ expression loss in prostatic adenocarcinoma was shown to result from an epigenetic effect – gene silencing caused by promoter methylation (Perry *et al*., 2007). Gene silencing due to promoter methylation provides an opportunity for clinical intervention, as gene-re-expression can be induced by a variety of DNA demethylating agents.

Apoptosis, i.e. natural programed cell death, is a physiological phenomenon indispensable for normal functioning of the organisms. The signal to apoptosis can be started practically in any cell. Disturbance in apoptosis regulation determines the essential link in the pathogenesis of many diseases, including cancer. Recent studies have shown, that genistein has multiple effects because it induces apoptosis also by mechanisms that do not involve ERs, as demonstrated in HepG2 cells, which do not express ERs (Chodon *et al*., 2007; Ørgaard and Jensen, 2008).

On trying to explain molecular mechanisms of apoptosis induction in human leukemia HL-60 cells by isoflavonoids from the leaves of *Milettia taiwaniana,* Murata *et al*. (2006) and Ito *et al*. (2006) found that isoflavonoids induced apoptosis through activation of the caspase-9/caspase-3 pathway which is triggered by mitochondrial dysfunction.

The biological mechanism by which phytoestrogens, particularly in the Western diet, can protect against hormone-dependent breast cancer is likely through their competitive effects on the generation, transport and removal of endogenous steroid hormones (Piller *et al*., 2006). By competing for estrogen receptors, phytoestrogens possibly inhibit binding of the more potent endogenous estrogens and decrease their potential effects on breast cancer risk (Verheus *et al*., 2007). They may inhibit also proliferation of hormone-independent breast cell lines via a number of mechanisms, including inhibition and down-regulation of protein tyrosine kinases, which are involved in growth signaling pathways. Genistein was shown to inhibit tyrosine kinase, particularly the autophosphorylation and activation of epidermal growth factor receptor, which is important in regulating apoptosis and cell proliferation. Genistein inhibited protein tyrosine kinase-dependent transcription of *c-fos* and subcellular proliferation in estrogen receptor negative human breast cancer cell lines. Key enzymes implicated in cancer invasion are also affected by genistein (Kousidou *et al*., 2006).

Changes in cell signaling were demonstrated in the HC11 mouse mammary epithelial cell line. Soy isoflavone genistein upregulated epithelial adhesion molecule E-cadherin expression and attenuated β-catenin signaling in mammary epithelial cells. In addition, it diminished basal and Wnt-1-(wingless)-induced cell proliferation and attenuated Wnt-1 targets *c-Myc* and c*yclin D1* expression (Su and Simmen, 2008).

## Mammographic density as a biomarker for breast cancer risk

Isoflavonoids have been increasingly advocated as potential natural alternatives to hormone replacement therapy. There are however only limited data on the effect of isoflavones on breast density, despite the fact that isoflavonoids were proved to be present in breast fluids and could directly act on breast tissue (Maskarinec *et al*., 2008a).

Although isoflavones have been suggested to protect against breast cancer, it is still not clear whether they act as estrogens or antiestrogens in breast tissue. Mammographic breast density, representing the amount of stromal and glandural tissue within the breast, has consistently been associated with a risk for breast cancer and a high density is often used as a predictive biomarker for breast cancer risk (Maskarinec *et al*., 2003). The mechanism underlying this relationship has not been fully explained, but it has been proposed that breast cancer density provides an index of current and past hormonal and reproductive events that modulate the risk for breast cancer. A high mammographic density has been associated with a 3- to 6-fold increase in breast cancer risk. Only few intervention studies with isoflavones and mammographic density have been published. In studies performed in premenopausal and postmenopausal women no effect of isoflavones from soy or other sources on mammographic density was shown (Maskarinec *et al*., 2003; Maskarinec *et al*., 2009). Neither did intervention studies with phytoestrogens from red clover or black cohosh show any statistically significant effects on mammographic density in postmenapausal women (Hirschberg *et al*., 2007). The findings in a 1-year double-blind, randomized, placebo-controlled trial did not support any effect (beneficial or adverse) of a large quantity of soy proteins containing isoflavones on mammographic density in postmenopausal women. The results of this trial do not support the hypothesis that a diet high in soy protein would decrease mammographic density in postmenopausal women (Verheus *et al*., 2008).

Although it has been hypothesized that breast density reflexes cumulative exposure to estrogens, in postmenopausal women the relationship between phytoestrogens and mammographic density may be different due to the decline of circulating levels of endogenous hormones after menopause. Yet examination of the relation between circulating sex hormones and mammographic density showed that in postmenapausal women mammographic density was not dependent on circulating sex hormone levels (Tamini *et al*., 2005; Elliassen *et al*., 2008).

Atkinson *et al*. (2004) did not observe a significant effect of clover-derived isoflavone supplement, taken daily for 1 year, on mammographic density in women aged 49–65 years, unlike conventional estrogen replacement therapies. Furhermore, they did not find any effect of the isoflavone supplement on estradiol, follicle-stimulating hormone or luteinizing hormone in postmenopausal women, or on hot flushes or other menopausal symptoms. A study aimed at soy isoflavonoid effects on endogenous estrogen metabolism in postmenopausal female monkeys showed that long-term exposure to soy isoflavonoids did not significantly alter gene markers of estrogen metabolism or estrogen receptor agonists in breast tissue. This long-term exposure may facilitate endogenous estrogen clearance and catabolism to more benign 2-hydroxylated metabolites (Wood *et al*., 2007).

Combined hormone replacement therapy may be associated with an increased risk for breast cancer and an increase in mammographic breast density. Breast cancer density increases when a woman starts on hormone replacement therapy and decreases when she discontinues it. Breast density is also reduced by antiestrogenic effect of tamoxifen, a selective estrogen receptor modulator.

Overall, recent epidemiologic and clinical data showed either a modest protective role or no effect of isoflavone intake on breast tissue density in pre- or postmenopausal women and on breast proliferation in postmenopausal women with or without a history of breast cancer (Messina and Wood, 2008).

## Modulation of immune function

The isoflavone daidzein administered orally stimulated murine nonspecific immunity, activated hormoral immunity, enhanced cell-mediated immunity, and at physiologically relevant concentrations potentiated lymphocyte activation, suggesting that its immunostimulatory effects may be involved in cancer prevention (Birt *et al*., 2001).

Equol is known to protect against solar-simulated UV radiation induced inflammation and immunosuppression. Two protective mechanisms are responsible: antioxidant actions and phytoestrogenicity. It is supposed that both might be functionally linked (Widyarini *et al*., 2006).

The role of the host immune function has become increasingly important in our understanding of the mechanisms involved in cancer prevention. The human immune system encompasses an array of defenses that help to guard against the development of age-related diseases, e.g. cancer. However, its function can be adversely affected by hormonal changes and oxidative damage (Kralova *et al*., 2008). The immune system may be compromised mainly after menopause because of the effects of aging and diminishing concentration of estrogen, an immune-modulation hormone. The human immune system may benefit from various biological properties of isoflavonoids. Since postmenopausal women are in particular susceptible to chronic disease associated with aging and to major shifts in hormonal status, isoflavones with estrogenic and antioxidant properties may offer immunologic benefits to women during this stage of life. Ryan-Borchers *et al*. (2006) reported that soy isoflavones stimulated an increase of important markers of immunity – B cells in healthy postmenopausal women.

Although soy isoflavones have been suggested to have both immune-enhancing and immune-suppressive effects, their effects on allergic disorders are less clear. Nagata *et al*. (2008) showed that a high intake of soy isoflavones was associated with decreased risk of cedar pollinosis, the most common seasonal allergic rhinitis.

## Effect on oxidative stress markers

Besides their estrogenic activities, isoflavonoids also display nonhormonal actions such as antioxidant effects. Antioxidant properties are one of the most important claims for food ingredients, dietary supplements and anticancer products. The antioxidant property of isoflavonoids offers an additional important mechanism through which they protect against chronic diseases.

Genistein and dadzein isolated from soybean showed stronger antioxidant activity than their glycosides, and dadzein isolated from *Pueraria lobata* exhibited the same antioxidant activity as α-tocopherol (Cherdshewasart and Sutjit 2008).

Cellular damage resulting from oxidative stress is supposed to be a major contributor to the etiology of cardiovascular disease through low-density lipoprotein (LDL) cholesterol oxidation and to the development of cancer through DNA strand breaks induction. The antioxidative efficiency of dietary isoflavonoids is associated not only with their reductive capacity but also with their protein-binding properties. In humans isoflavones may prevent low-density lipoprotein LDL cholesterol oxidation and lower markers of DNA oxidative stress *in vivo* (Ryan-Borchers *et al*. 2006). Since isoflavonoids can behave as antioxidants and thus protect against diseases resulting from oxidative damage, they may play a substantial role in disease prevention in postmenopausal women.

## Effect on vascular function

The postmenopausal status increases cardiovascular risk due to accelerated atherosclerosis progression. Prevention of intracellular lipid deposition may inhibit the formation of atherosclerotic lesions at the primary stage of atherogenesis at the level of the arterial wall. Although isoflavonoids may prevent atherosclerosis development in postmenopausal women, little is known about their direct effects on atherogenesis. Myasoedova and Sobenin (2008) showed that isoflavonoid-rich dietary supplement reduced the serum atherogenic potential in a double-blind placebo-controlled multicenter trial for the elucidation of anti-atheroslerotic activity of isoflavonoids.

Research on the effect of genistein on plasma nitric oxide concentrations, endothelin-1 levels and endothelium-dependent vasodilatation in postmenopausal women revealed that genistein therapy improved flow-mediated endothelium-dependent vasodilatation in healthy postmenopausal women. This improvement was mediated by a direct effect of genistein on vascular function and could be the result of an increased ratio of nitric oxide to endothelin (Sabudak and Guler, 2009).

## Effects on obesity; Regulation of adipogenesis

Experimental and epidemiological evidence has established an association between visceral obesity, a hallmark for the male obese phenotype, and metabolic syndrome, which retains its power throughout the spectrum of adiposity and is still clinically meaningful in severe obesity. The association may be due to an overload of liver free fatty acids produced by the high lipolitic activity of omental fat (Ørgaard and Jensen, 2008). Therefore men are usually at higher risk of cardiovascular disease than women (Stienstra *et al*., 2007). After menopause, the risk for women becomes similar to that for men because estrogen deficiency leads to visceral obesity, which is accompanied by a fall in insulin sensitivity and characterized by hyperglycemia and hyperlipidemia. Elevated blood lipid levels are closely associated with a very low-density lipoprotein VLDL and a low-density lipoprotein LDL cholesterol elevation and decreased levels of a high-density lipoprotein HDL cholesterol. E2 is a major regulator of adipocyte development and number in females and males. Due to structural similarities of isoflavones and E2, they can also influence the regulation of adipogenesis as a key process in obesity.

Moreover, increasing evidence has established that isoflavones not only act through the estrogen receptors but also exert effects through numerous other pathways, such as those regulated by the peroxisome proliferator-activated receptor (PPAR). Adipogenesis is then regulated by the hormonally induced cooperative interaction between members of the enhancer binding protein and peroxisome proliferator-activated receptor (PPAR) families and the primary adipogenic transcription factors. They act by synergistically transactivating the expression of several adipogenic effector genes. Unlike the highly specific ERs, PPARs bind a wide number of ligands and directly affect lipid metabolism by enhancing transcription of PPAR-regulated genes (Shen *et al*., 2006). The results of comprehensive studies have suggested that isoflavones may exert inhibitory effects on adipose tissue enlargement. But especially in humans, the actions of soy isoflavones appear to depend on a complicated interaction between many factors, such as the presence of soy protein and particular intestinal bacteria (Ørgaard and Jensen, 2008).

Despite the claims that link soy food consumption, through plasma cholesterol reduction, to a decreased risk of heart disease in humans, regular soy consumption did not significantly lower the LDL cholesterol in mildly hypercholesteromic subjects, both equol or nonequol producers (Thorp *et al*., 2008). On the other hand, research on isoflavones and their effect on obesity in cell cultures, rodents and humans, suggests that a high isoflavone intake with a high soy protein intake leads to significantly greater decrease in serum total and LDL cholesterol (not HLD cholesterol) than does low isoflavone intake, demonstrating that isoflavones have LDL cholesterol-lowering effects and improve blood lipid profiles, at least when consumed in combination with soy proteins (Ørgaard and Jensen, 2008).

## Effect of phytoestrogens on osteoporosis

Soy isoflavones may be useful in preventing and treating postmenopausal osteoporosis due to their similarity in structure to estradiol. They may thus act as potential replacements for estrogen deficiency. The implications of hormone replacement therapy in increasing the risk of heart disease and breast cancer have turned many women toward natural nutritional alternatives including phytoestrogens for the relief of menopausal symptoms and for preventive therapy of osteoporosis. Isoflavonoids and estrogens in hormone replacement therapies have overlapping yet sometimes divergent effects on the incidence of breast cancer and osteoporosis. Since stimulation of osteogenesis and concurrent inhibition of adipogenesis may explain the fact that estrogen deficiency decreases bone mass and increases adipose tissue, as seen in postmenopausal women, diets rich of isoflavonoids have positive effects on bone density and fracture rates (Ørgaard and Jensen, 2008). Isoflavonoids help to prevent osteoporosis by slowing down bone loss. Furthermore, several studies on soy supplementation and bone density suggest that soy products may be more effective in maintaining bone density in equol-producing individuals (Lampe, 2009).

As isoflavonoids are lipophilic, they can cross cell membranes and bind to cytoplasmic ERs, which proceed to act as transcription factors. This leads to increased gene expression of alkaline phosphatases and osteocalcin (bone formation factor), osteoprotegerin (an inhibitor of osteoclast activity) and IGF (a promoter of osteoblast activity) resulting in reduced bone resorption and increased bone formation (Lockwood, 2008).

Epidemiologic studies showed a positive association of soy intake and bone mass with increases in bone mineral density in the highest soy intake group. High consumption of soy isoflavones attenuated bone loss from lumbar spine of estrogen-deficient perimenopausal women. Erβ-specific targeting may produce beneficial effects in bones and may be applied as antiosteoporosis therapy in perimenopausal women (Chrzan and Bradford, 2007).

Daidzein was found to function as phytoestrogen in estrogen-responsive G-292 osterosarcoma cells. Transactivation studies with transfected ERs showed that this isoflavonoid was a potent activator of Erβ, though less potent than E2. Dadzein, like E2, inhibited IL-1β-cytokinin- and hormone-mediated IL6 (interleukin-6; a multifunctional inflammatory cytokine) secretion from G-209 osteosarcoma cell lines (Chrzan and Bradford, 2007). These results provide a basis for understanding how dietary phytoestrogens contribute to the osteoprotective effect without increasing the risk for breast cancer.
